# Effects of curcumin supplementation on the severity of intra-abdominal adhesions in rats

**DOI:** 10.1590/acb404425

**Published:** 2025-07-11

**Authors:** Pedro Afonso Kono, Rivair Gonçalves, Adriana Yuriko Koga, Melquesedeque dos Santos, Matheus Von Jelita Salina, Elder Dalazoana, Alceu Toledo, Leandro Cavalcante Lipinski, Marcos Ricardo da Silva Rodrigues, Eurico Cleto Ribeiro de Campos

**Affiliations:** 1 Universidade Estadual de Ponta Grossa – Departamento de Medicina – Ponta Grossa (PR) – Brazil.; 2 Faculdade Evangélica Mackenzie do Paraná – Departamento de Medicina – Ponta Grossa (PR) – Brazil.; 3 Universidade Estadual de Ponta Grossa – Departamento de Farmácia – Ponta Grossa (PR) – Brazil.

**Keywords:** Disease Models, Animal, Curcumin, Tissue Adhesions, Inflammation, General Surgery

## Abstract

**Purpose:**

To evaluate the effect of curcumin administered by gavage, on reducing intra-abdominal adhesions and attenuating the inflammatory process, assessed by a serum marker.

**Methods:**

Forty Wistar rats were randomly divided into two groups: the curcumin group, and the control group. The curcumin group received curcumin by gavage at the dose of 200 mg/kg over the seven days preceding and the seven days following surgery. In the control group, an isovolumetric administration of 0.9% saline solution was given by gavage. Both groups underwent a median laparotomy and left-sided colotomy. On the eighth postoperative day, the animals were euthanized for intracavitary adhesion analysis and left colon resection for histological examination. Intra-abdominal adhesions were classified from grade 0 to 4 based on increases in number, intensity, and ease of lysis.

**Results:**

Curcumin administration did not significantly reduce the severity of intra-abdominal adhesions (*p* = 0.7143) nor the severity of colonic inflammatory infiltration. However, a significant reduction in C-reactive protein levels was observed preoperatively in the curcumin group (*p* 0.05).

**Conclusion:**

While curcumin was not able to reduce the severity of intra-abdominal adhesions, it demonstrated the ability to attenuate the inflammatory process associated with the surgical procedure.

## Introduction

Intra-abdominal adhesions are abnormal fibrous bands located in the peritoneal cavity that connects organ surfaces that are normally separate^1^. The complications of peritoneal adhesions following surgical procedures have significant implications for patients, surgeons, and the whole healthcare system. Adhesions formed in the abdominal regions after abdominal or pelvic surgeries represent a natural response to peritoneal surface injuries during surgical procedures. These adhesions are associated with significant complications, such as adhesive small bowel obstruction, infertility in women, chronic abdominal issues, pain, and increased complexity in future surgeries^2^^,^ 3.

Peritoneal injury during surgery triggers inflammation and fibrin deposition in the peritoneal cavity. This, combined with coagulation imbalances following abdominal surgeries, leads to the formation of fibrinous exudates. Additionally, fibroblasts infiltrate in these fibrin deposits, generating a matrix rich in fibrocollagenous tissue, ultimately resulting in adhesions between intraperitoneal organs^4^.

Despite extensive research on the development of peritoneal adhesions, no definitive approach has been established to prevent their formation. This is due to ongoing controversies regarding the efficacy of currently available preventive agents^5^. For example, no pharmacological therapy has been approved for clinical use, as reported in the United States of America^6^.

Given this context, curcumin, a bioactive compound derived from *Curcuma longa* (turmeric exhibits anti-inflammatory, antioxidant, and chemopreventive properties in cancer treatment. When administered for seven days, curcumin has demonstrated antioxidant potential by reducing free radical-induced DNA damage. Through its anti-inflammatory and antioxidant effects, curcumin may have a beneficial role in reducing the severity of intra-abdominal adhesions^7^-^9^.

This study aimed to evaluate the effects of oral curcumin supplementation on the severity of abdominal adhesions in rats. Specifically, it sought to assess the clinical, biochemical, and histological effects of curcumin on abdominal adhesions in this experimental model.

## Methods

This study was an experimental animal model research conducted at the Laboratory of Operative Technique of the Universidade Estadual de Ponta Grossa (UEPG) between 2022 and 2023, following approval by the Ethics Committee on Animal Use of the UEPG (Protocol UEPG No. 23.000011573-2). All animals were housed in cages containing four individuals each, in a controlled environment with the temperature of 22 ± 1°C and regular photoperiod. Animal welfare monitoring and sample size calculation followed the guidelines of Normative Resolution No. 25 of the National Council for the Control of Animal Experimentation.

The total of 40 Wistar rats (*Rattus norvegicus albinus*, Rodentia, Mammalia), male, from a homogeneous lineage, were included in the study. The animals were obtained from the Animal Facility of the UEPG, Uvaranas Campus (Ponta Grossa, Paraná, Brazil). They were randomly distributed into two groups of 20 individuals each: a control group, and an experimental group treated with curcumin.

In the experimental group, curcumin was administered via gavage in the pre- and postoperative periods, covering seven days before and seven days after the surgical procedure, in addition to regular water and food. The curcumin dose used was 200 mg/kg, administered with an 8F tube (10 cm in length and 2 mm in internal diameter). To prepare the curcumin solution, 1,560 mg of turmeric with 96% curcumin concentration was diluted in 5 mL of glycerin, used to overcome curcumin’s low water solubility, along with 10 mL of 0.9% saline solution, resulting in a final concentration of 100 mg/mL. The daily dose was individually calculated based on the solution concentration (100 mg/mL), the animal’s body weight, and the target dose of 200 mg/kg. The curcumin was purchased from a certified laboratory with self-funded resources.

In the control group, gavage administration consisted of 0.9% saline solution at the dose of 2 mL/kg, following the same administration period as the curcumin-treated group. According to the guidelines of the Training in Ethics, Care, and Handling of Animals course offered by the Ethics Committee on Animal Use of the UEPG, sedation was not performed for the gavage procedure.

**Figure 1:**
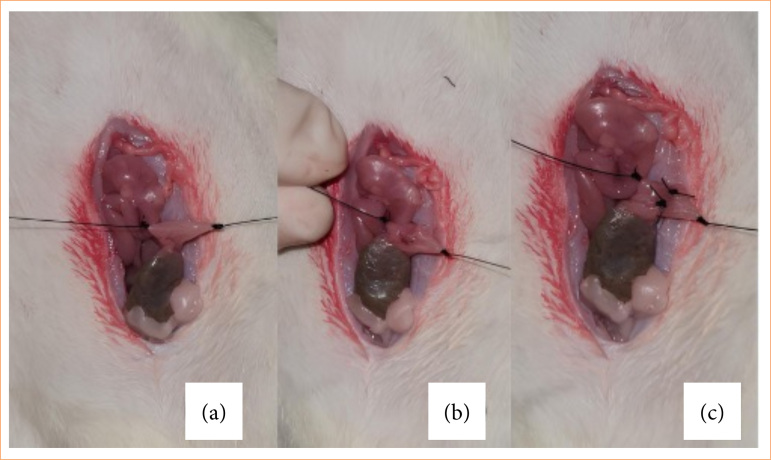
Surgical procedure inducing abdominal adhesions. **(a)** Left colon repair. **(b)** Colotomy of approximately 1 cm on the anterior wall of the left colon. **(c)** Anterior wall closure with two simple total sutures.

Source: Elaborated by the authors.

All animals underwent a 12-hour fasting period before the surgical procedure to reduce fecal content in the colon, facilitating the procedure and minimizing the risk of contamination. Anesthesia was administered intraperitoneally with ketamine at the dose of 40 mg/kg and xylazine at the dose of 10 mg/kg. After anesthetic induction, the animals were weighed, shaved, and underwent antisepsis. Access to the peritoneal cavity was obtained through a midline abdominal incision of approximately 3 cm, allowing exposure of the colon. The left colon was fixed (Fig. 1a), followed by a 1 cm colotomy on the anterior wall of the left colon (Fig. 1b). This experimental model was chosen due to its ability to mimic the high incidence of peritoneal adhesions observed in gastrointestinal surgeries10, as well as for its ease of standardization and reproduction by researchers.

Blood samples were collected from the inferior vena cava, after which the closure of the intestinal wall was performed in a single layer with two separate simple sutures using 5.0 monofilament nylon (Fig. 1c).

After anesthetic recovery, the animals received a single dose of fentanyl citrate (2 mg/kg) intraperitoneally for analgesia and were then relocated to cages, where they resumed access to water and food *ad libitum* 1 hour after the procedure. On the first postoperative day, curcumin administration was resumed in the experimental group. Throughout the pre- and postoperative periods, mortality was monitored, with subsequent investigation of the cause of death. Animals that died were excluded from the final sample analysis.

The rats were euthanized on the eighth postoperative day using an overdose of ketamine (160 mg/kg) and xylazine (40 mg/kg) via intraperitoneal injection. After euthanasia, the animals were weighed. A median thoracotomy was then performed, followed by right ventricular puncture to obtain 2 mL of blood from each specimen for biochemical analysis. The abdominal cavity was inspected through a median laparotomy to classify the severity of intra-abdominal adhesions. After classification, adhesions were lysed by dissection with hemostatic forceps, preserving the previous colon synthesis to avoid damage due to manipulation. Subsequently, the colon segment was resected for histological study.

**Figure 2:**
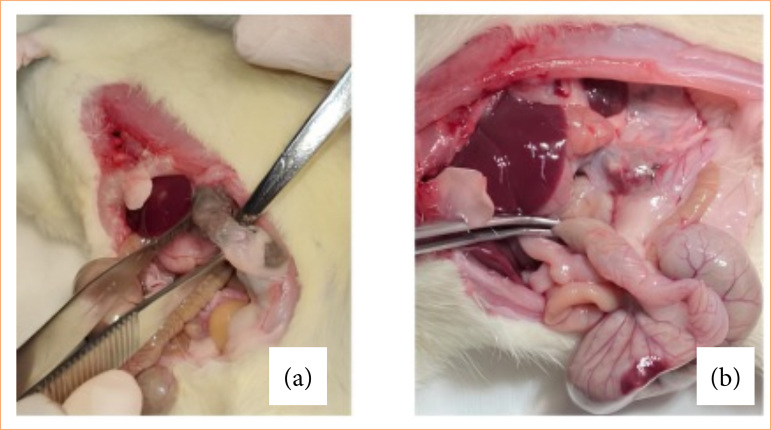
Classification of abdominal adhesions. **(a)** Grade 2 adhesion: small, weak adhesions that rupture with slight traction. **(b)** Grade 4 adhesion: numerous, extensive, and dense adhesions involving the mesentery, intestine, omentum, and abdominal wall.

Source: Elaborated by the authors.

During laparotomy, the abdominal cavity was inspected for the presence of adhesions, quantified according to the Knightly index^11^ (Table 1 and Fig. 2), widely used in studies on abdominal adhesions.

**Table 1:** Characterization of intra-abdominal adhesions.

Grade	Description
0	Absence of intra-abdominal adhesions
1	Single, thin, and easily separable adhesion
2	Small, weak adhesions that rupture with slight traction
3	Extensive visceral adhesions extending to the wall
4	Numerous, extensive, and dense adhesions involving the mesentery, intestine, omentum, and abdominal wall.

Source: Knightly et al.^11^.

**Figure 3:**
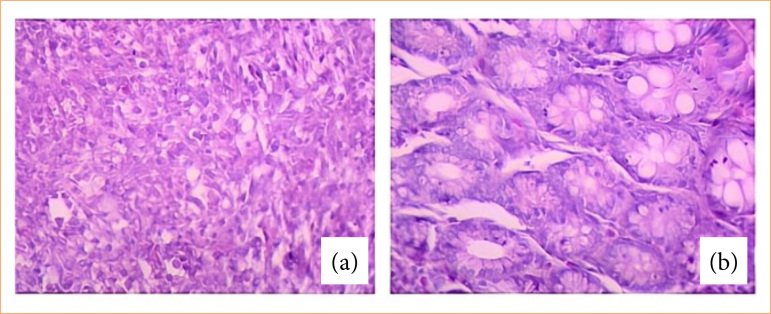
Histological analysis of the colon. **(a)** Grade 3 inflammatory infiltrate. **(b)** Grade 1 inflammatory infiltrate.

Source: Elaborated by the authors.

For the histological analysis of colonic mucosa, a semi-quantitative scoring system adapted from McCafferty et al.^12^ was used, based on two of the five analyzed characteristics. The selected characteristics included the extent of colonic mucosal architectural destruction (1: mild; 2: moderate; 3: extensive damage), visualized at 5x magnification, and the presence and degree of inflammatory infiltrate (1: mild; 2: moderate; 3: extensive infiltration), visualized at 20x magnification (Fig. 3). The histological slide images were obtained in a blinded manner.

During intraoperative procedures, 1 mL of blood was collected by inferior vena cava puncture to prevent hypovolemic shock and hypothermia, and 2 mL was collected by cardiac puncture post-euthanasia for C-reactive protein measurement.

Data were organized and analyzed using EpiInfo 7.2.4.0^®^ software. Descriptive analysis was performed using simple frequency (n) and relative frequency (%). All variables were tested for normality using the Kolmogorov-Smirnov test. Categorical variables were compared using the χ^2^ test and Fisher’s exact test, while continuous variables were analyzed using the Student’s t-test for normal data and the Mann-Whitney’s test for non-normal data. Normal variables were presented as mean, while non-normal variables were presented as median. *P* was considered significant when less than 0.05, with a 95% confidence interval.

## Results

Among the 40 rats available for the study, the final sample comprised 32 rats. One preoperative death occurred in the curcumin group due to complications related to gavage, while eight postoperative deaths were recorded—three in the control group and five in the curcumin group. These postoperative deaths were possibly attributable to difficulties in blood sample collection for biochemical analysis and prolonged exposure of the abdominal cavity, as indicated by the laboratory veterinary team. Consequently, analysis of the results was not feasible in eight rats due to the absence of adequate laboratory material. Therefore, the control group consisted of 17 rats (53.1%), while the curcumin group included 15 rats (46.9%).

In the control group, the mean preoperative weight was 261.1 g, and the postoperative weight was 267.7 g. In the curcumin group, the mean preoperative weight was 263.8 g, increasing to 273.2 g postoperatively. No statistically significant differences were observed between the groups.

Regarding C-reactive protein (CRP), the mean preoperative value was 0.094, decreasing to 0.06 postoperatively. In the preoperative comparison, the mean CRP in the control group was 0.112, while in the curcumin group, it was 0.06, demonstrating statistical significance (*p* < 0.05). However, no statistically significant differences were observed in postoperative CRP levels between the two groups (*p* > 0.05).

Table 2 presents the variables of weight, mortality, and CRP levels, demonstrating the homogeneity of the groups.

**Table 2:** Statistical analysis between groups. Comparison of mortality, pre- and postoperative weight, and C-reactive protein levels.

Variable	Total n (%)	Control n (%)	Curcumin n (%)	*p* -value
Death	8 (17.39)	3 (37.5)	5 (21.74)	0.3497*
Variable	**Total**	**Control**	**Curcumin**	***p* -value**
	Mean/median (SD); range	Mean/median (SD); range	Mean/median (SD); range	
Preoperative weight	262.4 (26.6); 216–326	261.1 (29.4); 216–326	263.8 (24.3); 232–314	0.753**
Postoperative weight	270.3 (32.3); 210–338	267.7 (31.1); 226–328	273.2 (34.6); 210–228	0.636**
Preoperative CRP	0.094 (0.027); 0.06–0.17	0.112 (0.025); 0.09–0.17	0.076 (0.014); 0.09–0.1	0.0001**
Postoperative CRP	0.06 (0.01); 0.05–0.1	0.06 (0.01); 0.05–0.09	0.06 (0.011); 0.05–0.1	0.7861***

* Fisher’s exact test;

** Student’s t-test;

*** Mann-Whitney’s test;

CRP: C-reactive protein.

Source: Elaborated by the authors.

Curcumin was not responsible for statistically significant changes in outcomes related to adhesion (*p* = 0.7143), architecture (*p* = 0.4519) or inflammatory infiltrate (*p* = 0.0954) of the colonic mucosa, when compared with the control groups. The variables related to the colon are described in Table 3.

**Table 3:** Statistical analysis between the groups. Comparison of the results of adhesions, inflammatory infiltrate and colon architecture between the control group and the curcumin group.

Variable	Category	Total n (%)	Control n (%)	Curcumin n (%)	*p* -value
Adhesion	Grade 1	2 (6.25)	1 (5.88)	1 (6.67)	0.7143*
	Grade 2	10 (31.25)	5 (29.41)	5 (33.33)	
	Grade 3	13 (40.63)	6 (35.29)	7 (46.67)	
	Grade 4	7 (21.88)	5 (29.41)	2 (13.33)	
Architecture	Grade 1	20 (62.5)	11 (55)	9 (75)	0.4519**
	Grade 2	12 (37.5)	9 (45)	3 (25)	
Inflammatory infiltrate	Grade 1	4 (23.53)	2 (16.67)	2 (40)	0.0954*
	Grade 2	5 (29.41)	3 (25)	2 (40)	
	Grade 3	8 (47.06)	7 (58.33)	1 (20)	

* χ^2^ test;

** Fisher’s exact test.

Source: Elaborated by the authors.

## Discussion

Adhesions, found in approximately 95% of patients undergoing abdominal surgery, can create technical challenges and increase the risk of complications. This includes difficulties in accessing the abdomen due to the loss of tissue landmarks or altered anatomy, as well as the potential for unintentional injury to abdominal organs, leading to increased healthcare costs^3^^,^ 13-15.

Regarding the pathogenesis of intra-abdominal adhesion formation, injury to peritoneal surfaces triggers a repair response that involves an inflammatory reaction, including cellular components and tissue-related factors, as well as the balance between coagulation and fibrinolysis^4^^,^ 16 ^,^ 17.

The literature has sought new treatments capable of reducing the incidence of adhesions after abdominal surgery. Physical barrier methods aim to isolate the injured peritoneum until re-epithelialization occurs. Although such methods limit the extent of adhesions, it is unclear whether they reduce associated complications^3^^,^ 18.

A meta-analysis of eight randomized clinical trials involving patients undergoing intestinal surgery revealed that physical barriers composed of hyaluronic acid sheets significantly reduced abdominal adhesions. However, there was no decrease in the incidence of postoperative intestinal obstruction (*odds ratio* [OR] 0.98, 95% confidence interval [95%CI] 0.78–1.23)^19^.

A systematic review of randomized clinical trials compared the use of glucocorticoids or promethazine with no treatment for preventing adhesions after gynecological surgery and found no evidence of a beneficial effect. In the same review, intraoperative peritoneal irrigation with steroids, dextran, or heparin was also evaluated, with no significant benefit observed^20^.

In the present study, curcumin did not show a significant impact on adhesion-related outcomes (*p* = 0.7143), architecture (*p* = 0.4519), or inflammatory infiltration of the colon (*p* = 0.0954). These findings suggest that, unlike other substances investigated in experimental models, curcumin may not exhibit the same preventive potential in peritoneal adhesion formation or inflammatory response modulation in the colon. Conversely, previous studies indicate that various pharmacological and natural agents have demonstrated efficacy in preventing these complications, highlighting the complexity of the inflammatory process and the need for compound-specific approaches^21^-^26^.

Kazemi et al.^22^ demonstrated that everolimus and prednisolone, both individually and in combination, significantly reduced adhesion formation in rats. These findings suggest that immunosuppressive and anti-inflammatory modulation plays a crucial role in preventing adhesions. The study underscores the potential of specific pharmacological agents targeting well-defined inflammatory pathways, something that curcumin—a compound with multiple molecular targets—may not replicate as efficiently in a specific experimental context.

Furthermore, Malekhosseini et al.^24^ evaluated the efficacy of colchicine, diphenhydramine, prednisolone, and their combinations in preventing intra-abdominal adhesions. The results indicated that colchicine, a well-known anti-inflammatory agent, showed promising outcomes. Comparing these findings with ours suggests that anti-inflammatory action alone may not be sufficient and that the specificity of the agent and therapeutic target is crucial for achieving positive clinical outcomes. In this regard, curcumin, with its general anti-inflammatory properties, may not be as effective as compounds directly targeting specific pathways involved in adhesion formation.

Beyond pharmacological interventions, studies such as that of Karaman et al.^21^ revealed that omega-3 fish oil, rich in anti-inflammatory fatty acids, was effective in preventing adhesions in rats. These results suggest that long-term dietary modulation can positively influence the inflammatory environment—an approach that, however, may not be replicated by acute or short-term curcumin administration in experimental models. This raises the possibility that the context of administration and exposure duration to compounds may be critical factors in the effectiveness of preventive strategies.

In studies focused on plant extracts, such as that of Raisi et al.^25^, which investigated the hydroalcoholic extract of *Salvia miltiorrhiza*, and that of Topal et al.^3^, which evaluated *Allium sativum*, both demonstrated inhibition of peritoneal adhesion formation in experimental models. These natural compounds, with antioxidant and anti-inflammatory properties, showed potential in preventing adhesions, suggesting that different bioactive compounds may offer advantages that curcumin, under the specific conditions of our study, failed to demonstrate. This also highlights the need to explore the complex interaction between natural compounds and the specific biological pathways involved in adhesion pathogenesis.

Finally, the study by Laukka et al.^23^ presented a different approach, using preperitoneal fat grafting as a physical barrier to prevent intra-abdominal adhesions. This mechanical method, in contrast to curcumin’s biochemical approach, suggests that physical or structural interventions may be more effective in certain contexts. While curcumin has shown multiple benefits in other contexts, our results indicated that its application as a preventive agent for adhesions may be limited, especially when compared to more specific or physical interventions.

In summary, the lack of efficacy of curcumin in our study, compared to the positive results of other compounds, underscores the importance of a diversified and specific approach in researching interventions for preventing peritoneal adhesions. These findings emphasize the need to continue investigating other substances and therapeutic combinations, as well as considering different administration strategies, to identify effective solutions that may eventually be applied in clinical practice.

Various studies have demonstrated the anti-inflammatory and antioxidant properties of curcumin, reducing free radical production and pro-inflammatory cytokines such as interleukin-8 and tumor necrosis factor-alpha. Additionally, curcumin inhibits the arachidonic acid cascade, playing a crucial role in treating various diseases^9^^,^ 25 ^,^ 27. Thus, the present study showed that curcumin’s anti-inflammatory effect was insufficient to reduce inflammation triggered by peritoneal injury and, consequently, to significantly reduce adhesion severity.

According to Isabella Luiz Suzuki^28^, “curcumin can freely pass through cell membranes due to its high lipophilicity (log *p* = 2.5). However, being an extremely lipophilic compound, the molecule has low water solubility (practically insoluble) and undergoes rapid degradation in the presence of light and aqueous media.” She also states that “the clinical advancement of curcumin formulations has faced challenges due to its low water solubility and short half-life, resulting in low bioavailability. Research has been conducted to find the best method for diluting curcumin in aqueous solution with acceptable stability and solubility”^28^. This justifies the limited impact observed in reducing intra-abdominal adhesion formation.

In this study, a statistically significant difference in CRP levels was observed between groups in the preoperative period (*p* < 0.05). However, in the postoperative period, no significant difference was found (*p* > 0.05). The reduction in systemic inflammatory response caused by curcumin in the preoperative period may be related to the attenuation of the endocrine and metabolic response to trauma. Since this response is triggered by surgery-related stress—ranging from emotional stress, pain, and anxiety in the preoperative period to more evident alterations in the postoperative period—, curcumin may have mitigated this process^29^.

With the activation of this response, there is an increase in pituitary hormone secretion and sympathetic nervous system activation, leading to increased acute-phase proteins and protein catabolism. Depending on the intensity of the trauma response and catabolism, numerous complications may arise, such as multiple organ dysfunction due to an exaggerated systemic inflammatory response^4^^,^ 29 ^,^ 30.

In the postoperative CRP analysis, the control group unexpectedly showed reduction in CRP levels, with a mean of 0.06, while the curcumin group maintained the same levels observed previously. This resulted in no statistically significant difference between the groups in the postoperative period (*p* > 0.05).

Therefore, rats supplemented with curcumin showed no benefit in abdominal adhesion formation or inflammatory infiltration in the colon. However, the curcumin group exhibited significant reduction in preoperative CRP, demonstrating that curcumin supplementation reduced the endocrine and metabolic response to trauma.

For future studies, we suggest increasing the sample size, using higher doses of curcumin, and prolonging administration. Additionally, other methods for inducing intra-abdominal adhesions should be considered to achieve more conclusive results.

Nonetheless, further research is necessary to prevent or mitigate adhesions, as no current method has demonstrated both reduced adhesion severity and decreased complications associated with intra-abdominal adhesions.

## Conclusion

Curcumin supplementation via gavage did not reduce the severity of intra-abdominal adhesions. However, in the curcumin group, there was a significant reduction in CRP levels during the preoperative period, indicating a potential benefit in modulating the endocrine-metabolic response to trauma.

## Data Availability

The data will be available upon request.
